# Mixed Methods Evaluation of Satisfaction with Two Culturally Tailored Substance use Prevention Programs for American Indian/Alaska Native Emerging Adults

**DOI:** 10.1007/s11121-023-01612-3

**Published:** 2023-11-04

**Authors:** Alina I. Palimaru, Ryan A. Brown, Daniel L. Dickerson, David Kennedy, Carrie L. Johnson, Elizabeth J. D’Amico

**Affiliations:** 1 RAND Corporation, 1776 Main Street, Santa Monica, CA 90401 USA; 2 UCLA Integrated Substance Abuse Program, Semel Institute for Neuroscience and Human Behavior, Los Angeles, CA USA; 3 Sacred Path Indigenous Wellness Center, Los Angeles, CA USA

**Keywords:** Substance use prevention/intervention, Emerging adults, Urban, Native American, Community-based participatory research

## Abstract

American Indian/Alaska Native (AI/AN) communities are disproportionately affected by the opioid epidemic. AI/AN emerging adults (ages 18–25) in urban areas are at particularly high risk, with the overdose death rate among urban-dwelling AI/AN people 1.4 times higher than rural-dwelling AI/AN people. Despite these challenges, there are no evidence-based culturally tailored prevention or intervention programs to address opioid, alcohol and other drug use among urban AI/AN emerging adults. This study focused on understanding AI/AN emerging adults’ experiences with two culturally tailored programs addressing opioid, cannabis, and alcohol use as part of the randomized controlled trial for Traditions and Connections for Urban Native Americans (TACUNA) in order to enhance feasibility of this intervention. Using a convergent mixed methods design at 3-month follow-up, we collected satisfaction and experience ratings and written narratives (total *n* = 162; intervention *n* = 77; control *n* = 85) from a sample of urban-dwelling AI/AN emerging adults who participated in both programs. We analyzed data through simultaneous examination of qualitative and quantitative data. The quantitative ratings show that both programs were rated highly. The qualitative data contextualized these ratings, illustrating pathways through which specific components were perceived to cause desired or observed behavioral change in participants. Among the elements that mattered most to these participants were the convenience of the virtual format, having a comfortable and safe space to share personal stories, and learning new information about their social networks. Negative comments focused on workshop length and inconvenient scheduling. This is one of the first studies to explore participant satisfaction and experience with culturally tailored substance use programming among a historically marginalized and understudied population. It is important to consider the voices of urban-dwelling AI/AN people in program development because hidden factors, such as limited financial resources, limited time, and misalignment with cultural values may prevent existing programs from being feasible.

**Supplementary Information:**

The online version contains supplementary material available at 10.1007/s11121-023-01612-3.

## Introduction

### Background

Communities across the USA have been affected by the opioid epidemic, with urban American Indian/Alaska Native (AI/AN) communities disproportionately affected. For example, AI/AN individuals had the second highest overdose rates from all opioids in 2017 (15.7 deaths/100,000 population) (Wilson et al., 2020). In addition, for this population, the overdose rate is 1.4 times higher in urban compared to rural areas (Joshi et al., 2018). According to the 2020 Census, 87% of those who identify as AI/AN alone or in combination live outside tribal lands, with 60% of that 87% based in metropolitan areas (HHS, 2022).

AI/AN communities have historically thrived in networks of immediate, extended, and communal families that play an important role in practical and spiritual support (Palimaru et al., 2022). This fabric of AI/AN life was undermined (directly and indirectly) by a combination of government-enforced relocations from tribal lands to urban areas; purposeful assaults against family, social, and cultural traditions; and other political and economic structural barriers that have fueled traumatic experiences and economic disenfranchisement for decades across multiple generations (Brave Heart & DeBruyn, 1998; Dickerson et al., 2020). Prior research found that historical and intergenerational trauma are key drivers of stressful and challenging social circumstances (Gibbs et al., 2018).

As a result of challenges at multiple levels of historical and social ecology, AI/AN emerging adults in urban areas face distinct and complex pressures around social and geographical fragmentation and limited opportunities for cultural involvement, which may in turn put some youth at risk for substance use (Besaw et al., 2004; Palimaru et al., 2022), poorer mental health (CDC, 2017) and death by suicide (Serfaini et al., 2017). Other reasons why AI/AN emerging adults may be vulnerable to substance use is the influence that occurs in their social networks, i.e., their families and peers, which may create normative pressure to take risks (Kennedy et al., 2022). Furthermore, national data show that alcohol and cannabis use are the most frequently used substances by emerging adults (Patrick et al., 2022). Likewise, our focus groups with AI/AN emerging adults, parents and providers in the development of this program highlighted the importance of not only addressing opioid use as part of the program, but also discussing how to make healthy choices around alcohol and cannabis (Dickerson et al., 2022).

### Framework for Program Development

In the face of these challenges, there are no evidence-based culturally tailored prevention programs to address alcohol and other drug use among urban AI/AN emerging adults (Venner et al., 2018). Given the understandable historical hesitation of some AI/AN communities to engage with established or US-government linked institutions and research projects, it is important to develop such programs using a community-based participatory approach (CBPR) (Crump et al., 2020; Gittelsohn et al., 2020; Whitesell et al., 2020). CBPR is a research approach centered on partnerships between scientific researchers and community members to investigate and address issues that affect minority communities disproportionately (Crump et al., 2020; Gittelsohn et al., 2020; Whitesell et al., 2020). The benefits of CBPR to AI/AN communities are multifaceted. For instance, CBPR can help strengthen community-level identity and capitalize on collective strengths (Israel et al., 1998; Walters et al., 2020). Collaborative partnerships across all stages of a study can also promote mutual learning and assist with revitalizing and preserving traditional culture and knowledge (LaVeaux & Christopher, 2010). Ultimately, one of the key benefits of aligning the rigors of research with community values and needs relates to building trust, which in turn could improve program implementation (Moran, 2001; Olson, 1999; Patel et al., 2022; Whitesell et al., 2020). In taking this approach, we worked with a community partner, Sacred Path Indigenous Wellness Center, to develop a culturally grounded opioid, cannabis, alcohol use prevention program for urban AI/AN emerging adults (D'Amico et al., 2021). We relied extensively on qualitative data and engaged throughout with our Elder Advisory Board and the broader urban AI/AN community (Dickerson et al., 2022).

In addition to drawing on quantitative data to develop interventions, it is equally important to capitalize on qualitative and mixed methods to evaluate intervention implementation. This helps ensure that interventions are continually responsive to community feedback. Qualitative data have often been poorly and inconsistently utilized in evaluation of randomized controlled trials, and few use a convergent approach as part of the evaluation to understand participants’ experience with the intervention and ways to improve it while the trial is ongoing (Davis et al., 2019). And yet, when used properly, qualitative data (such as participant narratives) can shed light on individual and contextual dynamics, and can help address many of the complex challenges that randomized control trials focused on preventive interventions face (Davis et al., 2019). Such information can help explain why programs do not achieve their full potential, and can be especially useful in adapting the design (Flemming et al., 2008) or cultural components of programs (Montgomery, 2016; Pallmann et al., 2018).

Much of the existing work in this area and for this population underscores the need to integrate traditional practices into prevention programming (Blue Bird Dickerson et al., 2020; Jernigan et al., 2020). For example, a previous study developed by our team (Motivational Interviewing and Culture for Urban Native American Youth, or MICUNAY) combined traditional practices with motivational interviewing to address substance use among urban AI/AN adolescents (Dickerson et al., 2016). At that time, we found that this approach helped promote resilience, and adolescents enjoyed the program (D’Amico et al., 2020; Dickerson et al., 2016). As a result, we took a similar approach to the development of the two prevention programs in this study (Traditions and Connections for Urban Native Americans and the Health and Wellness Program), by conducting formative focus groups, and building the intervention around motivational interviewing, which is a counseling method that includes careful listening and empowering discussions to encourage behavior change if adolescents are ready and willing (Miller & Moyers, 2017). The details of that development process, including how we used findings from focus group data to create program content are discussed at length in another manuscript (Dickerson et al., 2022). In addition, there is a significant body of work about the benefits of coupling social network visualizations with Motivational Interviewing in substance use prevention programs (Martinez et al., 2015; Rees et al., 2014; Tingey et al., 2016). Similarly, the feasibility and acceptability of incorporating social network visualizations into a culturally tailored motivational network intervention is described at length in a separate manuscript (Kennedy et al., 2022). The prior manuscript draws on formative focus group data before intervention implementation (collected from November 2019 to February 2020) to examine social network aspects as part of program development. This paper uses mixed methods and focuses on data from the 3-month follow-up after implementation of the two cultural programs (collected from April 2021 to July 2022) to understand social networks and other aspects of satisfaction from an implementation perspective.

This paper is one of the first to address participant satisfaction and experience during a randomized controlled trial among urban-dwelling AI/AN emerging adults. It is extremely important to consider the voices of this population in prevention program development, as hidden structural and socio-cultural factors, such as limited financial resources, competing demands on participant time, limited privacy, consequences of historical trauma, linguistic considerations, and misalignment with cultural values may prevent existing interventions from being feasible. We therefore describe pathways through which specific components of a culturally and developmentally tailored intervention can affect motivation for or actual behavioral change in participants. This study also provides a methodological advancement in the use of mixed methods to elicit early participant feedback on how to improve an intervention, by using joint displays of qualitative and quantitative data on similar topics. When used early during randomized controlled trials, this approach can help refine intervention design and implementation.

### Study Goals

This study focused on understanding AI/AN emerging adults’ experiences of two culturally tailored substance use prevention programs during the RCT so that we could utilize information to make improvements in format and content as needed. We focused on three research questions:

- How satisfied were participants with the workshops?
- How well did the workshops address mechanisms that prevent risk and enhance protection?
- What actionable recommendations for program improvement did participants suggest?

## Methods

### TACUNA Study

Traditions and Connections for Urban Native Americans (TACUNA) is a new opioid, cannabis, and alcohol use prevention program designed for urban AI/AN emerging adults. We are testing TACUNA as part of a longitudinal, mixed-methods clinical trial that draws on both quantitative (survey) and qualitative (focus groups, survey narrative elicitations) data (D'Amico et al., 2021). This study is comprised of two phases. Phase I focused on developing a culturally appropriate substance use prevention program that addressed opioid, alcohol, and other drug use (Dickerson et al., 2022). We are following a community-based participatory research (CBPR) approach, which is an equity-focused approach to the scientific process, with communities, researchers, and other stakeholders collaborating and partaking in the decision making and dissemination process (Crump et al., 2020). Phase II consists of a randomized controlled trial comparing the benefits of TACUNA to a culturally tailored control condition (D’Amico et al., 2021). For Phase I, we conducted 13 focus groups across California, involving 32 emerging adults, 33 providers, and 26 parents (Dickerson et al., 2022). Findings from focus groups are reported in detail elsewhere (Brown et al., 2022; Dickerson et al., 2022; Kennedy et al., 2022; Palimaru et al., 2022). Overall, these groups provided valuable information about the context of urban life, life challenges and aspirations, perceptions of social networks, and concrete ideas on how to execute the workshops. Our team tailored the workshop components based on these findings, for example adapting the cultural elements to preferences expressed by focus group participants (D'Amico et al., 2021). The initial plan for Phase II was to test in-person workshops in California. However, because of the COVID-19 pandemic, we shifted to a virtual format, which in turn allowed us to expand recruitment to urban areas across the USA.

TACUNA is comprised of three separate 2-h virtual workshops based on the Medicine Wheel, which is often included in traditional healing approaches (Dickerson et al., 2022). The first workshop focuses on healthy choices for the brain and a discussion of Native American identity; the second covers healthy choices for the body and Native American cooking; and the third workshop focuses on making healthy choices and improving spiritual health with a sage burning ceremony. See Table 1 for more details about what each workshop covered.

**Table 1:** Description of TACUNA workshops

**Workshop**	**Summary of activities**
1. Making healthy choices for my brain	Introduction, opening prayer, and purpose of group Generating rules for a successful group Discuss emerging adults’ AOD (alcohol and other drug) use Social networks and choices “Your Use” and choices Medicine Wheel Historical trauma and cultural identity Telling your own story (oral and digital)
2. Making healthy choices for my body	Introduction, opening prayer, and purpose of group Generating rules for a successful group Pros and cons of AOD use Social networks and choices The path of choices Willingness and confidence rulers around personal AOD use Medicine Wheel Discussion of Native American cooking with videos Cooking demonstration with virtual cooking for participants
3. Making healthy choices for my spirit	Introduction, opening prayer, and purpose of group Generating rules for a successful group What can happen when people use AOD Social networks and choices Thinking ahead and making healthy choices Wheel of the Future Willingness and confidence rulers around social network choices Medicine Wheel Discussion of spiritual life Smudging ceremony

Originally published in D’Amico, E. J., Dickerson, D. L., Rodriguez, A., Brown, R. A., Kennedy, D. P., Palimaru, A. I., Johnson, C., Smart, R., Klein, D. J., Parker, J., McDonald, K., Woodward, M. J., & Gudgell, N. (2021). Integrating traditional practices and social network visualization to prevent substance use: study protocol for a randomized controlled trial among urban Native American emerging adults. *Addict Sci Clin Pract*, *16*(1), 56

The TACUNA workshops included a novel social network component. To do this, we used a personal network interview platform, EgoWeb 2.0, an open-source survey software customized for social network data collection and visualization in interventions (see egoweb.info). Immediately after answering a series of questions about their social networks, participants in TACUNA viewed three visualizations of their network generated by EgoWeb 2.0. We also provided workshop participants with links to visualizations of their networks during the workshops. Workshop facilitators then used motivational interviewing, which is a goal-oriented style of communications that uses language focused on change (Miller & Rollnick, 2012), to generate group discussions about how social relationships relate to risk and resilience and help people make healthy choices in life (Kennedy et al., 2022). All workshops were piloted and refined based on feedback sessions that lasted approximately one hour (D'Amico et al., 2021). See Supplementary Fig. 1 for an overview of the social network visualization output.

For our control condition, we developed a 2-h culturally tailored opioid education workshop, hereafter referred to as the Health and Wellness Program. We included this active control condition for ethical reasons based on feedback from the community and our Elder Advisory Board. Specifically, the board felt that all participants should be given culturally appropriate programming relevant to opioids, in order to properly address the risks and disparities faced by AI/AN communities. The information in this workshop is based on prevention and education materials recommended by the National American Indian & Alaska Native Addiction Technology and Transfer Center, which is funded by the Substance Abuse and Mental Health Services Administration (NA-ATTC, 2019). The Health and Wellness Program differed in that it was more didactic and included a general overview of opioids, a discussion about the effects of the epidemic on AI/AN communities, as well as discussion of treatment options, physical wellness, and cultural traditions (D'Amico et al., 2021). See Supplementary Fig. 2 for an overview of the Health and Wellness Program content.

### Sample and Recruitment

Potential participants were eligible for TACUNA if they were: (1) between the ages of 18–25; (2) currently living in an “urban” area (i.e., not on a rancheria, reservation, or other tribal lands); (3) self-identified as AI/AN; (4) had no opioid use disorder; and (5) spoke English (D'Amico et al., 2021). This study occurred during the COVID-19 pandemic from December 2020 to October 2021; therefore, recruitment occurred online via social media across the USA, and participants completed surveys online. Participants completed an online screener, and those who were eligible were contacted by staff from our Survey Research Group and consented to be part of the study. They were then asked to complete a baseline survey and randomized to receive either one virtual workshop or three virtual workshops and a Wellness Circle (D'Amico et al., 2021). Procedures were approved by the institution’s Internal Review Board and the project’s Urban Intertribal Native American Review Board. This study has been preregistered with Clinical Trials, registration NCT04617938, and has published the study protocol (D'Amico et al., 2021). In addition to the baseline data, participants complete 3-, 6-, and 12-month follow-up surveys. The current analysis draws on the 3-month follow-up survey and open-ended comment data from AI/AN emerging adults across the USA who completed 3-month follow up surveys between April 2021 and July 2022 (total *n* = 162; TACUNA *n* = 77; Health and Wellness Program *n* = 85).

### Quantitative Data Collection

#### Demographics

Participants provided their age, gender, race/ethnicity, education level, and state of residence.

#### Alcohol, Cannabis, and Opioid Use

Separate items assessed number of times in the past 3 months participants reported drinking a full drink, 5 or more drinks (defined as heavy drinking), and using marijuana/cannabis or opioids (none, 1 time, 2 times, 3–5 times, 6–9 times, 10–19 times, 20–30 times, and 31 + times). More than half the sample reported alcohol use (77%), and cannabis use (52%) in the past 3 months. Close to half reported heavy drinking (48%). Few participants reported using opioids (2%).

#### Workshop Quality and Satisfaction

At the 3-month follow-up, participants were asked to rate the quality of the workshop they attended. Participants were asked, “How would you rate the quality of the workshops?” with answer options ranging from poor (1) to excellent (4). Satisfaction was measured with both an overall item (“Generally, I am satisfied with the workshop I attended”) and with a scale that included items about satisfaction with overall content, the workshop facilitator, learning new skills, understanding AOD use in one’s social network, and motivation to make changes to one’s social network (D’Amico et al., 2020). The answers ranged from strongly disagree (1) to strongly agree (5).

#### Peer Influence

We gauged peer influence on substance use by asking participants how much time they spent around others who use alcohol and other drugs (D'Amico et al., 2008). Questions asked “How often are you with people who are… (drinking alcohol, using marijuana, or smoking cigarettes) with response options from 0 (never) to often (3). In this paper, we focus on time spent with peers drinking alcohol, as it had one of the higher frequencies.

#### Cultural Identity

To assess AI/AN cultural identity, we used the Multigroup Ethnic Identity Measure (MEIM) (Phinney, 2016). The scale consists of 12 questions rated from 1 (strongly disagree) to 5 (strongly agree). For the purposes of our work with Indigenous communities, we modified MEIM items to focus on AI/AN heritage (e.g., “I have a clear sense of my AI/AN identity and what it means to me”) (Brown et al., 2019). For the mixed methods analysis we examined cultural themes along with the first item in the scale: “I have spent time trying to find out more about my American Indian/Alaska Native identity, such as its history, traditions, and customs,” which was the item that was closest conceptually to the themes with which it was overlaid in the mixed methods analysis (see Stage 2 analysis below).

#### Qualitative Data Collection

We supplemented quantitative data with six open-ended questions about participants’ workshop experience for both conditions: “Please describe how you feel about your experience”; “What did you like most?”; “What did you like least?”; “How might you improve the workshops?”; “How did you feel about the virtual experience?” and “How did you feel the workshops addressed your experiences as an urban Native American young adult?”.

In addition, TACUNA workshop participants answered four open-ended questions addressing the Social Network component: “Please describe what you thought about seeing the picture of your social network and the discussion of social networks”; “How did the discussion of social networks help you think about drug and alcohol use in your own social network?”; “How did seeing the social network visualization and the discussion help you understand traditional practices and Native American culture in your social network?”; and “Describe any changes you made to your social network, or relationships to people in your network, that were the result of seeing the visualization and the discussion.”

There were no character limits to comment length that participants could write in response to any question.

### Mixed Methods Analysis

Both qualitative and survey data were uploaded to NVivo, a mixed methods software for coding and organizing survey and qualitative data (QSR, 2018).

#### Stage 1: Qualitative Analysis

First, we conducted manifest content analysis on all text responses to open-ended questions, using 30 codes focused on experiences during the workshops, 10 codes focused on the social network visualization and discussion, and 15 codes describing actionable recommendations for improving the intervention (Kleinheksel et al., 2020). Codes were developed inductively (Cho & Lee, 2014) by one person (first author), and reconciled with another team member (second author). Both coders were trained in qualitative methods in the context of health services research and anthropology; and both have considerable prior experience with the methodology employed, the subject matter, and Indigenous communities. Thus, the analytic process may have occasionally drawn on assumptions and expectations associated with prior work. Neither coder is AI/AN, however both participated in the formative focus groups as moderators, and have previous experiences partnering with AI/AN communities across the USA to ensure the research process reflects community traditions, values, and preferences. Furthermore, numerous discussions were held with the entire research team regarding these data, including author DLD, who is a Native American addiction psychiatrist working in the Native American community, and author CLJ who is CEO of our community partner SPIWC, who is also Native American and has worked with AI/AN communities for over two decades.

In some cases, we developed codes based on the topical focus of each question. For example, many of the comments in response to the question “How did you feel about the virtual experience?” were coded with a Virtual Format parent code, with relevant subcodes captured under that code, such as “technical challenges,” “liked virtual format overall,” and “disliked virtual format overall.” The same applies for the question about recommended improvements. For broader questions, such as “How did the discussion of social networks help you think about drug and alcohol use in your own social network?”, the codes were based entirely on the comments, which include themes focused on network size, impact of network relationships, isolation, and so on.

We applied some codes to multiple questions, because sometimes answers went beyond the scope of the immediate question. For instance, participants described changes to their social networks in response to the prompt “Describe any changes you made to your social network, or relationships to people in your network, that were the result of seeing the visualization and the discussion.” But content about network changes also occurred in response to other questions, such as “How did seeing the social network visualization and the discussion help you understand traditional practices and Native American culture in your social network?” Some codes contained content that was exclusively positive in valence, some were exclusively negative, and others had both positive and negative content. Also, the experience codes and the suggestions for improvement codes are separate because we did not necessarily want to implicate respondents into recommended changes on basis of experience comments, especially as they were given as part of a distinct prompt for improvement suggestions. Given that narratives were rich, and that we coded segments of text that were sufficiently long and coherent to be interpretable on their own, some segments were assigned multiple codes. The full codebook is available online as a Technical Supplement.

#### Stage 2: Analysis of Themes by Survey Answers

Next in the analysis, we followed a “convergent” mixed methods approach wherein we examined qualitative experiential themes sorted by categorical survey ratings (Creswell, 2015; Fetters, 2019). The convergent approach was chosen because it would provide multiple pictures of the concept of interest, i.e., satisfaction, from several angles. Gauging only closed-ended ratings would preclude narrative content about dimensions of experience that may relate to participant satisfaction ratings but are not captured with the survey questions. Likewise, only relying on narratives may not exhaust all the dimensions of satisfaction within the closed-ended scale. This convergent approach allowed the authors to iterate and draw “meta-inferences,” i.e., to find linkages between qualitative and quantitative data, and to interpret both types of data relative to each other (Creswell, 2015; Fetters, 2019).

## Results

In total, 162 respondents provided ratings to the survey items (TACUNA *n* = 77; Health and Wellness Program *n* = 85), of whom 152 provided at least one comment.

### Demographic Characteristics and Other Descriptive Information

Table 2 summarizes demographics for both TACUNA and Health and Wellness Program participants. Overall, participants were 18–26 years (mean = 22.2, SD = 2.19) and were predominantly female (85%). Ninety-eight percent of participants (all but two) identified as AI/AN. Of those 160 participants, 42% endorsed AI/AN alone, 32% identified as AI/AN in combination with Hispanic ethnicity (and in some cases an additional racial category as well), and 26% endorsed AI/AN plus another race (but not Hispanic ethnicity). These racial and ethnic categories are consistent with Census 2020 data where respondents identified as AI/AN alone or in combination, and with prior evidence (Brown et al., 2016). We do not provide tribal affiliation to protect participant confidentiality.

**Table 2:** Sample Demographics (N = 162)

	**Total** **(N = 162)**	**TACUNA** **(N = 77)**	**HWP** **(N = 85)**
**Age range (Mean)**	18–26 (22.2)	18–26 (22.2)	18–26 (22.2)
**Sex N (%)**			
Male	23 (14%)	10 (13%)	13 (15%)
Female	138 (85%)	67 (87%)	71 (84%)
Decline	1 (1%)	-	1 (1%
Race/Ethnicity N (%) *			
American Indian/Alaska Native (only)	67 (42%)	30 (33%)	37 (44%)
Asian/Pacific Islander	1 (1%)	1 (1%)	-
Black	1 (1%)	-	1 (1%)
Hispanic (in combination with AI/AN)	51 (32%)	23 (30%)	28 (33%)
Multiracial (AI/AN plus any two or more races)	42 (26%)	23 (30%)	19 (22%)
**Education N (%)**			
Less than High School	3 (2%)	3 (4%)	-
HS eq/GED	3 (2%)	2 (3%)	1 (1%)
HS grad	85 (52%)	33 (43%)	52 (61%)
AA	17 (10%)	12 (16%)	5 (6%)
Bachelors	41 (25%)	21 (27%)	20 (24%)
Professional degree or certification	7 (4%)	3 (4%)	4 (5%)
Masters	6 (4%)	3 (4%)	3 (4%)

* racial and ethnic groups do not add up to 100% as participants could report “all that apply.” TACUNA stands for the intervention group, Traditions and Connections for Urban Native Americans. HWP stands for the control group, Health and Wellness Program

More than half of respondents graduated from high school and nearly a third had a Bachelor’s degree. The two groups were comparable with regards to age and education, with no statistically significant differences. Participants resided in 22 different states. Eighty-six percent of participants in each group provided comments in response to the open-ended questions.

### Satisfaction and Quality Ratings

We present descriptive information on satisfaction and quality ratings in Table 3. Within a range of 1 to 4, the mean quality rating for TACUNA was 3.2, with 81% rating it as “excellent” or “good.” In the Health and Wellness Program group, the mean quality rating was 3.1, as 79% rated it “excellent” or “good.” From a range of 1 to 5, the average satisfaction rating of TACUNA participants was 4.34 (77% “somewhat agreed” or “strongly agreed” with the statement), and 4.45 for the Health and Wellness Program group (83% agreed “somewhat” or “strongly”).

**Table 3:** Proportion of respondents by quality and satisfaction ratings

	**TACUNA** **(N = 77)**	**HWP** **(N = 85)**
Quality*		
How would you rate the quality of the workshop(s)?	**81%**	**79%**
Satisfaction**		
Generally, I am satisfied with the workshop(s) I attended	**77%**	**83%**
Therapeutic alliance**		
The workshop leader(s) and I respected each other	82%	85%
The workshop leader(s) respected my background (e.g., age, gender, race, culture, ethnicity, sexual orientation, disability, lifestyle, etc.)	79%	88%
The workshop leader(s) helped me believe that I could change and improve my life	65%	72%
I have learned skills to help me to manage my life better	71%	70%
The workshop leader(s) was/were helpful	77%	82%
I would recommend these workshops to a friend	77%	82%
Session style**		
I felt the workshop leader(s) respected where I was with my alcohol and/or drug use and that any change was up to me	76%	78%
The workshop leader(s) valued my opinion	79%	84%
The different activities we did together in the workshops were helpful	76%	80%
I learned more about Native American culture	75%	67%
Perceived empowerment**		
I feel that the things I did in the workshops will help me to make the changes that I want	70%	71%
I could use information from the workshops in my daily life	77%	80%
I could understand the information from the workshops	82%	87%
I developed new friendships as a result of participating in the workshops	38%	35%
The workshops helped me better understand the connections between alcohol, drug use, and people in my social network	70%	74%
The workshops helped me better understand the connection between traditional practices and people in my social network	68%	69%
The workshops inspired me to make changes to my own social network	55%	61%
Participating in the TACUNA cultural activities can help me lead a healthier life	74%	n/a
I enjoyed the discussion of social networks	69%	n/a
I enjoyed seeing pictures of my social network	62%	n/a

^*^For quality, percent reflects participants who reported excellent or good

^**^For satisfaction, therapeutic alliance, session style, and perceived empowerment, percent reflects those who reported “strongly agree” or “somewhat agree”

*TACUNA stands for the intervention group, Traditions and Connections for Urban Native Americans. HWP stands for the control group, Health and Wellness Program*

### Quality Ratings Matched Diverse Qualitative Experiences

Table 4 lists the proportion of participants across both positive and negative themes. Most participants liked the virtual format (primarily due to its convenience) and enjoyed learning new information. TACUNA participants also indicated they enjoyed meeting and connecting with AI/AN emerging adults, whereas Health and Wellness Program participants did not mention this theme. Participants from both groups felt it was a comfortable and safe space to share their views and felt validated in their experiences. Several other positive dimensions of satisfaction were present only for the TACUNA group, such as appreciating the traditional practice and the cultural grounding of the content.

**Table 4:** Positive and negative themes among participants (N = 152)

**Positive themes**	**% TACUNA participants**	**% HWP participants**
Enjoyed virtual format	89	84
Enjoyed learning new information	50	77
Enjoyed meeting and connecting with AI/AN emerging adults	39	n/a
Comfortable and safe space to share	36	34
Felt validated	27	10
Addressed urban experience	26	30
Overall positive comments on workshop	26	23
Traditional practice	24	n/a
Cultural grounding	17	n/a
Facilitator	12	n/a
Liked video component	n/a	16

**Negative themes**	**% TACUNA participants**	**% HWP participants**
Did not like virtual format	27	43
Lengthy duration	25	20
Inconvenient scheduling	22	n/a
Group was too small	13	20
Limited opportunities to interact	13	14
Prior familiarity with topic	8	n/a
Too much interaction	6	n/a
Inability to connect with participants afterwards	4	n/a
Limited participation from others	4	n/a
Inadequate pace	2	5
Did not address Native experience	n/a	20

*TACUNA stands for the intervention group, Traditions and Connections for Urban Native Americans. HWP stands for the control group, Health and Wellness Program*

Negative themes related to not enjoying the virtual format (mostly because of technical challenges), and some felt the workshops were too long. Also, some participants commented on inconvenient scheduling. Negative comments also indicated that some groups were perceived to be too small, with limited opportunities to interact with others.

Table 5 displays quality ratings along with the three most common positive themes and illustrative examples, both for the TACUNA workshop and the Health and Wellness Program. Of all TACUNA participants who offered both ratings and comments (*n* = 66), 49% rated it as “Excellent” and 43% rated it as “Good.” Among these, 92% said they enjoyed the virtual format, writing, for example that: *“It was convenient, since I didn’t have to go anywhere far for it and it made the length of it more manageable.”* Thirty-nine percent felt TACUNA was a comfortable and safe space, writing, for example that *“I feel it was a great safe space to talk about the experiences I have dealt with growing up as an urban native.”*

**Table 5:** Overall satisfaction themes by workshop quality ratings

% Talked about theme	Theme	Representative quote
Of all TACUNA Workshop participants who offered both ratings and comments (*n* = 66), 49% rated it as “Excellent” and 43% rated it as “Good”		
92%	Enjoyed virtual format	*“It was convenient, since I didn’t have to go anywhere far for it and it made the length of it more manageable.”*
52%	Learn new information	*“Learning more about sage and food. Also learning more about the medicine wheel.”*
39%	Comfortable and safe space to share	*“I feel it was a great safe space to talk about the experiences I have dealt with growing up as an urban Native.”*
Of all HWP participants who offered both ratings and comments (*n* = 73), 37% rated it as “Excellent” and 47% rated it as “Good”		
74%	Enjoyed virtual format	*“I greatly enjoyed the virtual workshop and was grateful for the caution that it applied to prevent the spread of Coronavirus.”*
67%	Learned new information	*“I’ve had a good experience with everything including the workshop over zoom where we talked about opioids and the various effects of the drug and how hard it is to stop doing an opioid due to the overwhelming withdrawal cycle.”*
32%	Comfortable and safe space to share	*“I felt more connected, given that each person was most likely in a space that they were already comfortable in, and with this it provided a sense of safeness and allowed the discussions be more open and really went in-depth about the topics.”*

*TACUNA stands for the intervention group, Traditions and Connections for Urban Native Americans. HWP stands for the control group, Health and Wellness Program*

### Social Network Awareness Motivated Change

Table 6 lists the proportion of TACUNA participants who mentioned social network themes along with quotes that illustrate how the workshops addressed mechanisms that prevent risk and enhance protection inherent in social relationships. These themes were mentioned only by the TACUNA participants, because only they received the social network component. Notably, more than half of respondents (53%) indicated that they understood how their social network relationships influenced their alcohol and other drug use, as well as participation in traditional practices. They also described either real or desired changes in their social networks (52%).

**Table 6:** Proportion of TACUNA participants with Social Network-specific themes

Social network themes	Percent of workshop participants who commented (*n* = 66)	Illustrative quotes
Understood nature and impact of social network relationships	53	*“It showed me that I need to build stronger boundaries with myself since I am influenced by my network a lot.”*
Real or desired changes in their social networks	52	*“It showed me that I didn’t have a lot of people in my network who participated in traditional practices and that I need to step out of my comfort zone to seek that out.”*
Understood network composition	42	*“It showed me that there was a lot of users in my network.”*
No changes to social network	41	*“I haven't made any changes, there are no AN people around me to befriend. In the future I want that to change, and I want more AN friends.”*
Liked the visualization	39	*“I was surprised because I had never seen a diagram like that before and it put my life and relationships into perspective.”*
Realized socio-cultural isolation	27	*“I realized that I did not have many people in my social network that practice traditional culture, and that may be the reason why I sometimes feel disconnected from my culture.”*
Did not learn anything new regarding social network	26	*“I already knew the only people in my social circle who participated in cultural practices were family members, so the chart was not very helpful.”*
Discussion was informative or helpful	17	*“It was very effective and informative.”*

Table 7 displays social network themes by the frequency of being around people who drink alcohol. Of all TACUNA participants who offered ratings (*n* = 65), 25% were “often” and 45% were “sometimes” around people who are drinking. Of these, almost half (49%) described real or desired changes to their networks, as this comment illustrates: *“The visualization helped me think in the future about my choices of who I am hanging out with and more specifically what we are doing. I am more interested in doing activities and things sober and want to try to bring that to my friend groups.”* Thirty-one percent of TACUNA participants were “hardly ever” or “never” around people who are drinking. Of these, 60% were motivated to make real or desired changes to their network; for example, *“I changed my social network by hanging out with different people and expanding my friend groups, but also drifted away from some friends.”*

**Table 7:** Overall Social Network themes by frequency of being around people who are drinking

% Talked about theme	Theme	Representative quote
Of all TACUNA Workshop participants who offered both ratings and comments (*n* = 65), 25% were “Often” and 45% were “Sometimes” around people who are drinking		
49%	Understood nature and impact of SN relationships	*“It mapped clear connections between social circles, activities, and interests that gave me a sense of why some people in my social circles were more likely to use substances. It also gave me a good picture of who was sober, and why they were more connected.”*
49%	Described real or desired changes to their social network	*“The visualization helped me think in the future about my choices of who I am hanging out with and more specifically what we are doing. I am more interested in doing activities and things sober and want to try to bring that to my friend groups.”*
42%	Understood network composition	*“I noticed that a lot of artists in my social network use alcohol and that since many Native people I knew through ceremony, they were less likely to use substances.”*
Of all TACUNA Workshop participants who offered ratings and comments (*n* = 65), 31% were “Hardly ever” or “Never” around people who are drinking		
65%	Understood nature and impact of SN relationships	*“It helped me by making me realize that I was responsible for myself and to know to not let those who may use substances influence me.”*
60%	Described real or desired changes to their social network	*“I changed my social network by hanging out with different people and expanding my friend groups, but also drifted away from some friends.”*
45%	Understood network composition	*“Helped me realize few people in my life use drugs, but the ones I care about most are susceptible.”*

### Culturally Adapted Segments Validated Urban Native Experience

Among TACUNA participants, 39% said they liked the workshops because they were able to meet and connect with other AI/AN emerging adults; 27% felt validated in their experiences as young Native American people; 26% felt that TACUNA addressed their urban experience; and 24% enjoyed the traditional practice components. Others appreciated TACUNA’s cultural grounding (17%), and 8% were motivated to learn about their culture, often through reaching out to their community.

Table 8 displays prominent culture and identity themes for the majority of TACUNA participants — those who indicated they have sought information on Native identity. Of all TACUNA participants who offered both ratings and comments (*n* = 54), 57% “Strongly Agreed” and 43% “Agreed” with the statement “I have spent time trying to find out more about my AI/AN identity, such as its history, traditions, and customs.” Of these, 47% appreciated that they were able to meet and connect with other AI/AN emerging adults during the workshops, as illustrated by this quote: *“I loved the opportunity to speak with other Indigenous young adults about topics that aren’t easily brought up.”* Thirty-five percent suggested they felt validated: *“I like hearing what other AI/AN [emerging adults] have to say about their experiences, too. It helped validate my feelings on identity and what it means to have traditional practices in an urban setting.”*

**Table 8:** Prominent culture and identity themes by pursuit of information on Native identity among TACUNA participants

% Talked about theme	Theme	Representative quote
Of all TACUNA Workshop participants who offered both ratings and comments (*n* = 54), 57% “Strongly Agreed” and 43% “Agreed” with the statement “I have spent time trying to find out more about my AI/AN identity, such as its history, traditions, and customs.”		
47%	Meet and connect with young AI/ANs	*“I loved the opportunity to speak with other indigenous young adults about topics that aren’t easily brought up.”*
35%	Felt validated	*“I like hearing what other AI/AN have to say about their experiences too. It helped validate my feelings on identity and what it means to have traditional practices in an urban setting.”*
29%	Enjoyed traditional practice	*“I loved the workshops!! It was my first time really getting to practice my culture rather than read about it online or in a book. Even when I worked on a reservation I did not get as much cultural access as I did through these workshops. I wish there were more spaces like this where I could learn about my heritage without worrying about judgement.”*
29%	Addressed urban experience	*“It is important because it is a reality for many urban Natives.”*
22%	Cultural grounding	*“The workshops were grounded by culture.”*
10%	Motivated action on culture	*“It made me realize I need to embrace and grow through learning and practicing my cultural heritage.”*

### Actionable Recommendations for Improvement

Table 9 shows the proportion of participants by each improvement theme. Nearly a fifth of the TACUNA participants suggested improving facilitation techniques and increasing participant interaction. For example, one respondent wrote, *“I would improve the workshops by maybe doing more icebreakers so it does not feel as awkward and there is a greater sense of connection with the other participants.”* Another suggested, *“I feel there should be discussion questions given to us where we can talk amongst ourselves regarding how we may improve our Native community.”*

**Table 9:** Improvement themes among participants (N = 152)

**Themes**	**% TACUNA (*n* = 50)**	**% HWP (*n* = 45)**	**Representative quotes**
Improve facilitation techniques	18	7	*“I would say having the facilitators be open to answering the questions themselves and talking about their personal experiences, if comfortable, since it felt more like a lecture than an open dialogue between two people.” (TACUNA)* *“The way the speakers deliver information. No room for real discussion.” (HWP)*
More participant interaction	18	20	*“More breakout rooms to facilitate interactions.” (TACUNA)* *“Time with Native peers in discussion.” (HWP)*
More Native cultural content	16	13	*“I know traditions for Plains tribes are very different than, say, those on the East coast, so maybe I would take the time to elaborate on that.” (TACUNA)* *“I wanted to hear more about the difficulties of being a reconnecting Native.” (HWP)*
Offer better scheduling options	12	2	*“More weekends workshops or earlier workshops during the week.” (TACUNA)* *“Make it more accessible for people with various schedules.” (HWP)*
Improve pace	10	4	*“I may improve it by not letting the silence go on too long or spending too much time on a topic.” (TACUNA)* *“Maybe send the videos out for people to watch before hand.” (HWP)*
Shorter duration	10	11	*“Maybe make them shorter. It was a bit hard for me to attend them because of my schedule.” (TACUNA)* *“Maybe make the workshop more concise, taking maybe 45 min to an hour.” (HWP)*
More engaging content	8	13	*“Include more activities and videos. I really enjoyed all of them.” (TACUNA)* *“Add games or something fun.” (HWP)*
Larger groups	6	20	*“Have larger groups than just the three plus one moderator so that there could be more discussion in the meeting.” (TACUNA)* *“I would have added more people if possible.” (HWP)*
Offer in-person options	6	4	*“I think the only thing that could’ve made these workshops any better would be the opportunity to meet in person.” (TACUNA)* *“I think in-person would have been nice.” (HWP)*
Longer workshop	4	7	*“When we talked, time felt short.” (TACUNA)* *“Allow the meeting to be a little longer.” (HWP)*
Smaller groups	4	4	*“Keep the workshops a little smaller. The smaller groups made for more participation.” (TACUNA)* *“Smaller groups could be more informal and focus on getting to know participants.” (HWP)*
Address technical challenges	2	7	*“Not everyone has good wifi service. I wish the cameras would stream everyone’s faces.” (TACUNA)* *“Train staff on how to work the platform they are on.” (HWP)*

*TACUNA is the Intervention, Traditions and Connections for Urban Native Americans. HWP is the Control, Health and Wellness Program*

Sixteen percent also recommended more tailored Native cultural content, as illustrated by the following quote: “*I would improve the workshops by breaking down information by region. I know traditions for Plains tribes are very different than say those on the East coast, so maybe I would take the time to elaborate on that.”*

Among Health and Wellness Program participants, 20% recommended having larger groups and more participant interaction; for example, “*I would include more areas to discuss the material, case studies *etc*. to dive more deeply into the material. Also, larger groups to interact if possible.”*

## Discussion

This study describes urban AI/AN emerging adults’ satisfaction with two culturally tailored programs addressing opioid, cannabis and alcohol use. Results from this community-based study highlight the importance of analyzing satisfaction levels and feedback from participants during the randomized controlled trial. This feedback can help to address implementation issues early in the research process, which can help with community-based delivery of interventions and development of interventions that can be applied nationwide. It is important to consider participants’ voices as they can reveal hidden structural and socio-cultural factors that may undermine program effectiveness. This study also uses mixed methods to elicit early participant feedback on how to improve the intervention, by using joint displays of qualitative and quantitative data.

We utilized a convergent mixed-methods design to elicit actionable information about implementation, feasibility, and acceptability. The quantitative ratings show that both programs were rated highly, and the qualitative data helped contextualize ratings, to understand how the programs worked. We expected that participants would report high satisfaction with both programs as content was developed with extensive input from the community. Methodologically, this study shows the utility of garnering both quantitative and qualitative satisfaction and experience data early on in randomized control trials, which can flag implementation issues early. For instance, even participants who rated the TACUNA workshop highly offered suggestions for improvement regarding workshop duration, size, and scheduling convenience. Without qualitative data, such important actionable details might have been missed.

Overall, participants in this study reported high satisfaction levels with both interventions. Participants liked the convenience of the virtual format, the comfortable and safe space to share personal stories, and learning new information. The narratives also provided insights on mechanisms that prevent risk and enhance protection. Participants in the TACUNA workshops reported that the social network component helped raise awareness of their own social networks, inspired motivation to change their social networks, and inspired motivation to connect to culture. Participants’ comments illustrated how seeing illustrations of their social networks helped them think about who was around them, how they interacted with others, whether they needed to make changes, find support, or take other action. Respondents also noted the importance of the cultural practice components, saying they enjoyed learning about traditional practice and history, with some signaling motivation to connect with the community more. Overall, findings help substantiate our approach of incorporating social network discussions and AI/AN traditional practices within the TACUNA program.

Moreover, the qualitative data offered actionable information regarding implementation, such as the positive regard for the virtual format and requests for more regionally focused traditional information. We found pathways through which specific components of TACUNA were perceived to increase motivation for or actual behavioral change in participants. Our team has used these comments to further enhance implementation of the intervention. For example, responding to negative comments about the duration of the intervention, we reduced the workshop length from two to 1 h. We used the findings relating to perceptions of facilitators in our facilitator training sessions, for example to help better pace the sessions. We also plan to enhance the final manual and intervention approach to reflect the need for more local cultural information. For example, prefacing the Native American cooking component with historical overviews of Native plants and local or regional preferences for seeds and other ingredients.

Finally, our work reinforces the importance of using community-based participatory research throughout the entire study. Many of the 3-month respondent observations in this study aligned with insights from our formative focus groups and pilot tests, wherein emerging adults indicated that the social network component was helpful as it created an understanding of how their networks may influence them, and many felt motivated to make healthy connections (Brown et al., 2022; Kennedy et al., 2022; Palimaru et al., 2022). We actively engaged members of the community at key steps along the way, including the design and content of the intervention, ensuring culturally-appropriate recruitment, and dissemination of results (Dickerson et al., 2022). This is especially important in under-represented communities that have faced historic abuses in the name of research.

There are a few limitations to note. First, recall bias may be an issue, as participants responded 3 months after the intervention. The narratives included occasional comments such as *“I don’t know”* or *“I don’t remember.”* Moving forward, study designs that examine satisfaction and experience at multiple points in time, such as immediately after intervention and at 3 months, could offer more insights into the optimal time to elicit such feedback. Also, we had a slightly smaller qualitative sample compared to the survey ratings sample. There were respondents who answered the closed ended questions, but not the open-ended qualitative questions. Finally, a majority of our sample reported female identity; this aligns with prior findings in prevention research, showing that females typically have higher participation rates (Reed et al., 2022). Thus, these findings may overrepresent female perspectives and sensitivities relating to substance use and social networks, while underrepresenting other gender identities.

## Conclusion

This is one of the first studies to examine participant satisfaction and experience with substance use prevention programming among a historically marginalized population. This study elicited actionable information about feasibility and acceptability of two culturally tailored programs that were developed through community-based participatory research. Overall, findings highlight the importance of engaging communities throughout the intervention development process as part of a continuous dialogue on how to ensure programs are relevant and grounded in community priorities and needs. Collecting and analyzing participant ratings and narratives during the implementation process provided a deeper understanding of the workshops, including successful and less helpful aspects, which can aid in future development and refinement of programs.

### Supplementary Information

Below is the link to the electronic supplementary material.

Supplementary file1 (DOCX 157 KB)

Supplementary file2 (DOCX 599 KB)

## Data Availability

The data used to support the findings of this study are available on request from the corresponding author. The data are not publicly available due to privacy or ethical restrictions.
