# Supplementary material for: Mixed Methods Evaluation of Satisfaction with Two Culturally Tailored Substance use Prevention Programs for American Indian/Alaska Native Emerging Adults
Source: Prev Sci. 2023 Nov 4;25(2):330–46. doi: 10.1007/s11121-023-01612-3 (PMC10891245; doi:10.1007/s11121-023-01612-3)
Supplement: Supplementary file 1 — Supplementary file1 (DOCX 157 KB) [file 11121_2023_1612_MOESM1_ESM.docx]

Supplemental Figure 1. Network visualizations auto-generated using EgoWeb


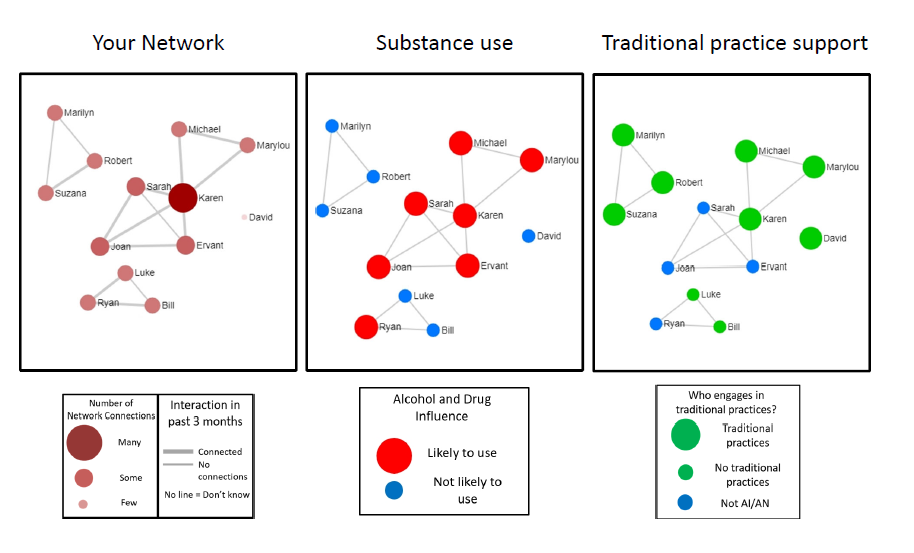


Note: Network members are represented by circles (nodes), and lines between nodes represent network contacts who interacted with each other in the past two weeks. “Your Network” visualization shows the names of people the participant reported interacting with in the past two weeks and highlights the centrality of nodes by calibrating node size and color with degree centrality (number of connections for a particular node), and line thickness with the participant’s rating of relationship strength between the two nodes. “Substance use” shows larger red nodes for people who the respondent rates as likely to use AOD in the next two weeks and smaller blue nodes for those who are unlikely. Finally, “Traditional Practice Support” shows larger green nodes for people who engage in traditional practices, and smaller blue nodes for people who do not.

Originally published in D'Amico, E. J., Dickerson, D. L., Rodriguez, A., Brown, R. A., Kennedy, D. P., Palimaru, A. I., Johnson, C., Smart, R., Klein, D. J., Parker, J., McDonald, K., Woodward, M. J., & Gudgell, N. (2021). Integrating traditional practices and social network visualization to prevent substance use: study protocol for a randomized controlled trial among urban Native American emerging adults. *Addict Sci Clin Pract*, *16*(1), 56.
