# Supplementary material for: Mixed Methods Evaluation of Satisfaction with Two Culturally Tailored Substance use Prevention Programs for American Indian/Alaska Native Emerging Adults
Source: Prev Sci. 2023 Nov 4;25(2):330–46. doi: 10.1007/s11121-023-01612-3 (PMC10891245; doi:10.1007/s11121-023-01612-3)
Supplement: Supplementary file 2 — Supplementary file2 (DOCX 599 KB) [file 11121_2023_1612_MOESM2_ESM.docx]

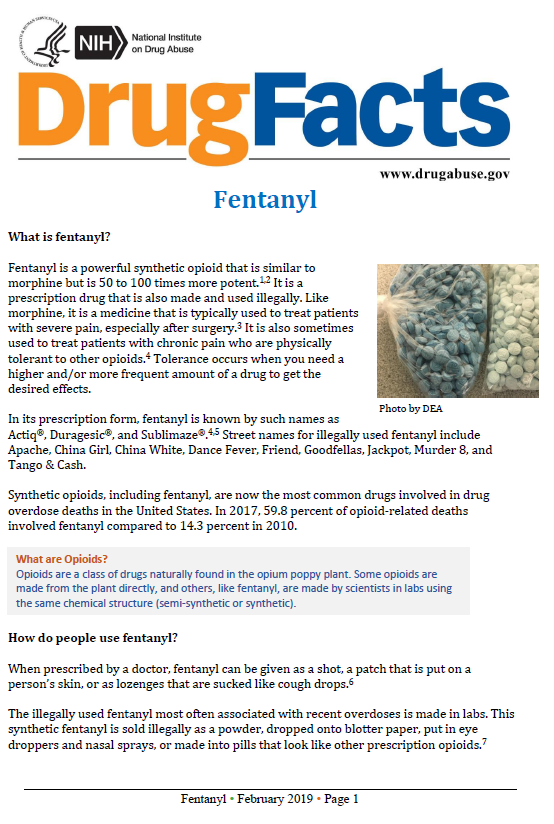
Supplemental Figure 2a. Opioid education workshop, general overview of opioids


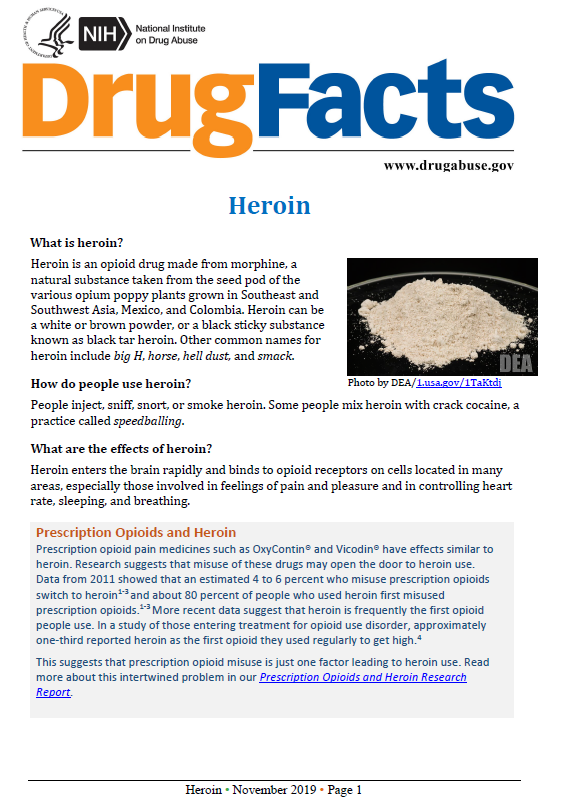


Originally published in D'Amico, E. J., Dickerson, D. L., Rodriguez, A., Brown, R. A., Kennedy, D. P., Palimaru, A. I., Johnson, C., Smart, R., Klein, D. J., Parker, J., McDonald, K., Woodward, M. J., & Gudgell, N. (2021). Integrating traditional practices and social network visualization to prevent substance use: study protocol for a randomized controlled trial among urban Native American emerging adults. *Addict Sci Clin Pract*, *16*(1), 56.

Supplemental Figure 2b. How the opioid epidemic has affected AI/AN communities


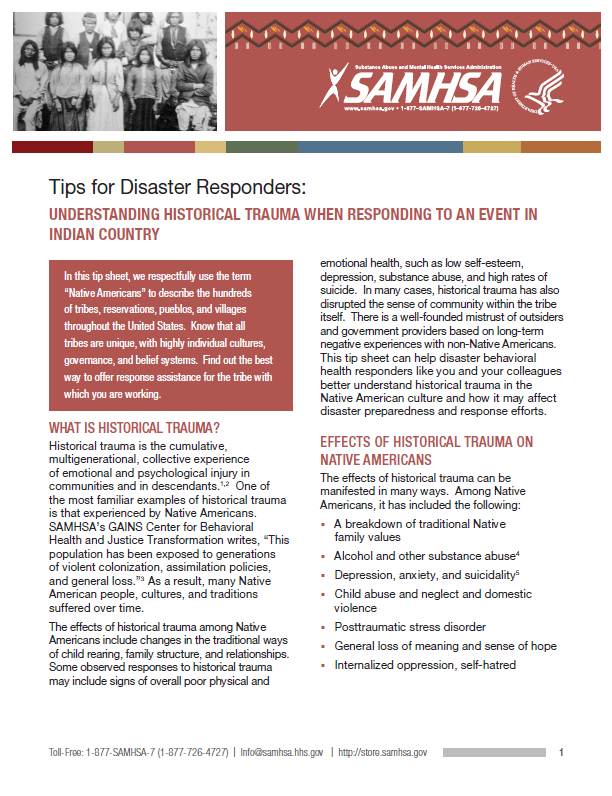


Originally published in D'Amico, E. J., Dickerson, D. L., Rodriguez, A., Brown, R. A., Kennedy, D. P., Palimaru, A. I., Johnson, C., Smart, R., Klein, D. J., Parker, J., McDonald, K., Woodward, M. J., & Gudgell, N. (2021). Integrating traditional practices and social network visualization to prevent substance use: study protocol for a randomized controlled trial among urban Native American emerging adults. *Addict Sci Clin Pract*, *16*(1), 56.
